# Simultaneous Preparation of Salidroside and *p*-Tyrosol from *Rhodiola crenulata* by DIAION HP-20 Macroporous Resin Chromatography Combined with Silica Gel Chromatography

**DOI:** 10.3390/molecules23071602

**Published:** 2018-07-02

**Authors:** Liwei Sun, Ran Zhou, Jinling Sui, Yujun Liu

**Affiliations:** 1 National Engineering Laboratory for Tree Breeding, College of Biological Sciences and Biotechnology, Beijing Forestry University, Beijing 100083, China; lsun2013@bjfu.edu.cn; 2 School of Nature Conservation, Beijing Forestry University, Beijing 100083, China; zhouransha@163.com

**Keywords:** salidroside, *p*-tyrosol, DIAION HP-20 adsorption column chromatography, silica gel column chromatography, UPLC

## Abstract

The *Rhodiola* species have a long history of utilization in traditional medicine and have been considered as a source of adaptation to environmental challenges; salidroside and *p*-tyrosol are the major responsible compounds. Here we propose a novel UPLC-guided two-step method consisting of a DIAION HP-20 adsorption and silica gel column chromatographies, which can simultaneously prepare high purities of salidroside and *p*-tyrosol with noticeable yields from the rhizome of *Rhodiola crenulata*. Results demonstrated that DIAION HP-20 could successfully remove all impurities except crenulatin during a gradient elution with 5–20% ethanol, which could achieve an optimal purification of salidroside and *p*-tyrosol with increasing rates of 29.19% and 33.44%, respectively. Furthermore, chloroform was selected as an ideal solvent for separating *p*-tyrosol with salidroside, and thus crenulatin was subsequently applied in the silica gel chromatography, and the separation of salidroside with crenulatin could be achieved using silica gel chromatography with a mixture of chloroform and methanol at a volume ratio of 4:1. High purity rates of 94.17% and 97.29% and overall yields of 39.09% and 43.73% for salidroside and *p*-tyrosol were simultaneously achieved. Our method provides a new way to simultaneously obtain salidroside and *p*-tyrosol from *R. Crenulata*, as well as other related plant species.

## 1. Introduction

The *Rhodiola* species have been known as a traditional medicine in Asian and eastern European countries to maintain overall health and are considered to be a source of adaptation to environmental challenges. It has been used as an adaptogen in people with high intensity work as well as in some special professions including athlete, mountaineer, pilot, and astronaut to improve the body’s ability to adapt to adverse circumstances [1,2]. In recent years, these species have been reported to show beneficial properties, such as myocardial protection [3], antidepressant [4], immune enhancing [5], anti-stress effects [6], and hepatoprotective activities [7]. In addition, *Rhodiola* species have also been reported to improve work productivity and effect memory and learning [8,9], as well as to retard the aging process [10].

**Figure 1:**
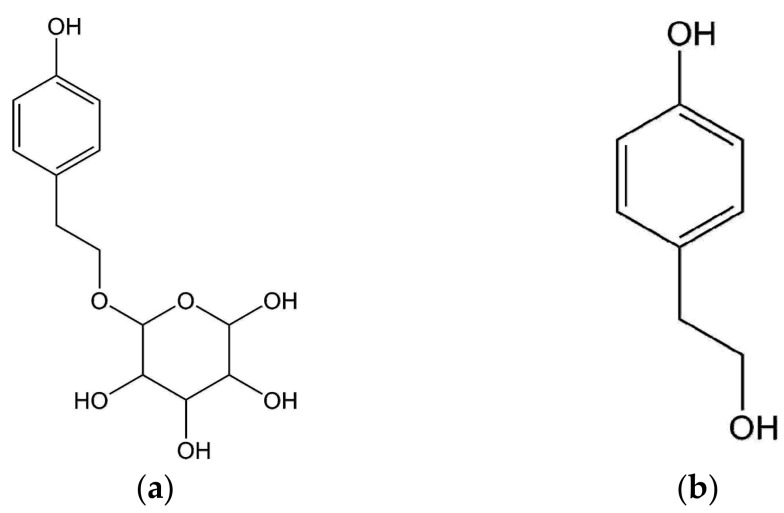
Chemical structure of salidroside and *p*-tyrosol. (**a**) salidroside; (**b**) *p*-tyrosol.

Salidroside (Figure 1a), 2-(4-hydroxyphenyl) ethyl-β-D-glucopyranoside, has been affirmed as the main responsible ingredient in the *Rhodiola* species [2,11]. It has been reported to possess a variety of pharmacological activities such as anti-cancer [12], anti-hypoxia [13], anti-oxidant [14], anti-aging [15], and protection against ischemia/reperfusion injuries on the myocardium [3]. *p*-Tyrosol (Figure 1b), 2-(4-hydroxyphenyl)-ethanol is another potent phenolic compound responsible for the *Rhodiola* species [2,11]. Documents have shown that *p*-tyrosol deliver functions including the antiadipogenic effect [16], cardioprotection against I/R injury [17], and improve overall oxidative stress in various human tissues [18,19,20]. Therefore, as the principal active ingredients in the *Rhodiola* species, the levels of salidroside and *p*-tyrosol commonly serve as markers of medicinal quality for the *Rhodiola* species. Among the *Rhodiola* species, *Rhodiola crenulata* has been officially designated as the main medicinal species in the Chinese Pharmacopeia [21], in which levels of those two compounds are high [22]. Because of such beneficial effects delivered by salidroside and *p*-tyrosol, mounting attention has been focused on the preparation of these two essential compounds. Although documents have shown that salidroside and *p*-tyrosol can be generated through certain methods such as enzymatic synthesis [23] or plant tissue culture [24], the most convenient and direct source to obtain salidroside and *p*-tyrosol until now remains the extract from the plant. Many efforts have been made to develop methods for the extraction of salidroside and *p*-tyrosol; however, only a few reports have covered the purification and subsequent separation.

Some reports have documented that new technologies, including high-speed counter-current chromatography, can prepare a salidroside with high purity, however, complicated and costly mixed phase solvent systems were applied, including ethyl acetate-n-butanol-water (1:4:5, *v*/*v*) and chloroform-methanol-isopropanol-water (5:6:1:4). However, the yield was disappointingly low [25]. Alternatively, a new method has been developed that adsorbs column chromatography, it has the advantage of low operating costs and a high regeneration rate. However, it is difficult to separate compounds with close polarity and thus its application is restricted as a result. In fact, only 13.8% (*w*/*w*) of salidroside was reached in the product purified by the DA-201 adsorption resin [26]. Although there was literature showing that the repeated performance of adsorption chromatography can obtain salidroside with a relatively higher purity [27], it still cannot fit into simultaneous preparation of salidroside and *p*-tyrosol. Therefore, concerning the simultaneous obtainment of these two compounds from the *Rhodiola* species, standard methods such as liquid-liquid extraction, polyamide column, as well as silica gel chromatography, still appear to be the most promising technologies by far. Ioset et al. [28] reported that the polyamide column can remove polymeric polyphenols from low-molecular weight compounds in *Rhodiola rosea* rhizomes. However, currently there is a lack of documentation for the preparation of salidroside or *p*-tyrosol by the standard method, not to mention their simultaneous preparation, thus it is a challenge and demands prompt investigation.

In this study, we explored a combination of new and standard methods based on adsorption chromatography combined with silica gel chromatography in order to simultaneously prepare salidroside and *p*-tyrosol from *R. crenulata* extract. An efficient method was established and these two compounds were obtained in high purities and with noticeable yields.

## 2. Results and Discussion

### 2.1. Enrichment of Salidroside and p-Tyrosol from RCE with DIAION HP-20 Adsorption Chromatography

#### 2.1.1. Screen of Eluent(s) for the DIAION HP-20 Adsorption Chromatography

**Figure 2:**
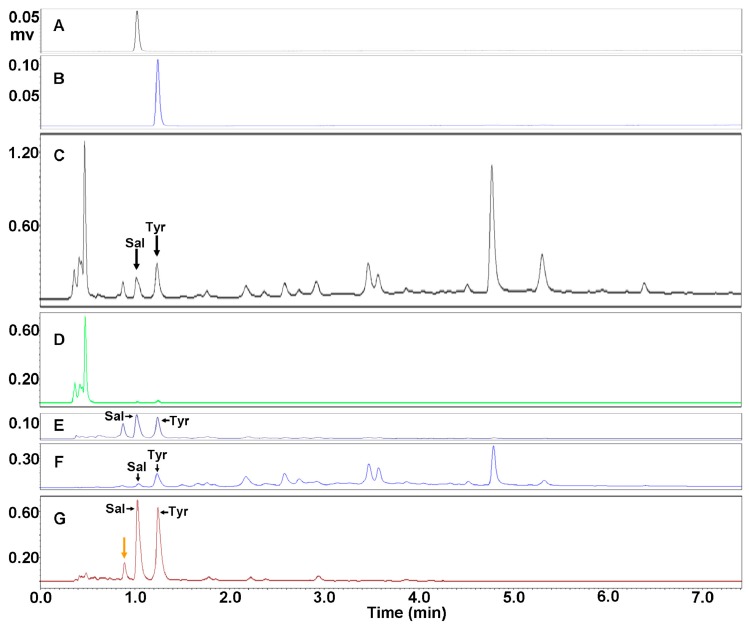
UPLC profiles for purification of salidroside and *p*-tyrosol with DIAION HP-20 adsorbent resin. Shown in sequence are profiles of salidroside standard (**A**), *p*-tyrosol standard (**B**), *R. crenulata* crude extract (RCE) (**C**), water eluent (**D**), 15% ethanol eluent (**E**), 25% ethanol eluent (**F**), and fraction enriching salidroside and *p*-tyrosol, i.e., FEST (**G**). Ordinates of (**C**–**G**) were proportionally adjusted for comparison. Sal and Tyr refer to salidroside and *p*-tyrosol, respectively.

Through ultrasonic extraction, *R. crenulata* crude extract (RCE) was obtained by three repeats of 75% methanol extraction after the removal of lipid-soluble impurities via a petroleum ether extraction. Concentrations of salidroside and *p*-tyrosol reached 2.25% and 3.36% (Table 1), which were observed at 1.05 and 1.25 min, respectively, as validated by their standards (Figure 2A–C).

In order to increase concentrations of salidroside and *p*-tyrosol, DIAION HP-20 adsorption resin chromatography was then applied, and enriching eluent(s) were firstly screened. This started with the fractionation of RCE by aqueous ethanol from low to high concentrations, and eluents of water, and 5%, 10%, 15%, 20%, 25%, and 50% ethanols were orderly obtained. All eluents were then subjected to UPLC to examine the concentrations of salidroside and *p*-tyrosol. Figure 2D–F shows UPLC profiles of water, 15% ethanol, and 25% ethanol eluents, which were close to (or the same as) the 5% ethanol, those of both 10% and 20% ethanols, and that of the 50% ethanol, respectively.

**Figure 3:**
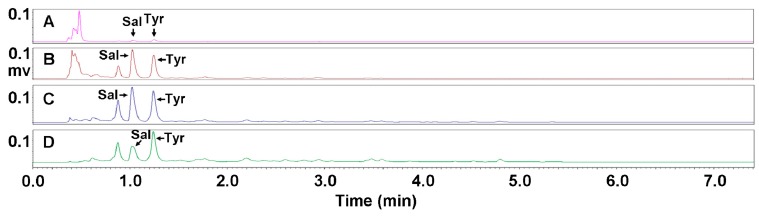
UPLC profiles for the purification of the salidroside and *p*-tyrosol by 5%, 10%, 15%, and 20% ethanol with DIAION HP-20 adsorbent resin. Shown in sequence are profiles of 5% ethanol eluent (**A**), 10% ethanol eluent (**B**), 15% ethanol eluent (**C**), 20% ethanol eluent (**D**). Sal and Tyr refer to salidroside and *p*-tyrosol, respectively.

As shown in Figure 2D, only a large and wide multi-peak represented by the highest peak at 0.5 min existed, demonstrating that eluents of water and 5% ethanol mainly enriched water-soluble impurities with high polarity. The difference is that salidroside and *p*-tyrosol were hardly observed in the profile of the water eluent, while in that of the 5% ethanol eluent these two compounds presented both minute peaks (Figure 3A), indicating that the 5% ethanol began to enrich the salidroside and *p*-tyrosol.

Salidroside and *p*-tyrosol were mainly presented in UPLC profiles as represented by that of 10%, 15% and 20%, ethanol eluent in Figure 3B–D, demonstrating that these two compounds were concentrated in eluents of 10%, 15%, and 20% ethanol. Through detailed comparisons between the UPLC profiles of these three eluents, it was found that the ratio of salidroside/*p*-tyrosol was initially high, and then turned out to be low with the increase of ethanol concentration from 10% to 20%. This indicates that eluents of 10% and 15% ethanol enriched more salidroside than *p*-tyrosol, while the 20% ethanol better concentrated the *p*-tyrosol. Among these three eluents, the 15% ethanol was shown to be the best for purifying the salidroside and *p*-tyrosol, and the concentrations of salidroside and *p*-tyrosol reached 21.51% and 20.28%, respectively (see Figure 2E and Figure 3C; Table 1).

In addition, Figure 2F presented many peaks behind *p*-tyrosol as well as minor salidroside and *p*-tyrosol, implying that the enrichment of salidroside and *p*-tyrosol from RCE could be completed before the 25% ethanol eluent, and could successfully separate these two compounds with other peaks behind. Therefore, it can be concluded that the ethanol concentration from 5% to 20% could be capable of separating the salidroside and *p*-tyrosol from the peaks behind the *p*-tyrosol via DIAION HP-20 adsorption resin chromatography.

#### 2.1.2. Enrichment of Salidroside and *p*-Tyrosol by the DIAION HP-20 Adsorption Chromatography

Based on the above finding, the method for enrichment of salidroside and *p*-tyrosol by DIAION HP-20 adsorption chromatography was established, which started from water and 5% ethanol washing in sequence, followed by a gradient elution of 5% to 20% ethanols. The results are shown in Figure 2G and Table 1. Salidroside and *p*-tyrosol were enormously presented in the UPLC profile (Figure 2G), and concentrations of salidroside and *p*-tyrosol further increased to 31.44% and 36.80%, respectively (Table 1, FEST, i.e., elutions No. 1–100 that correspond to 5–13.33% of ethanol elution), which were superior to those of the 15% ethanol eluent (21.51% and 20.28%, respectively). These results indicated a good concentrating effect by this method. Furthermore, statistical significance was found between the fraction enriching of salidroside and *p*-tyrosol (FEST) and RCE, confirming that this method was effective for the enrichment of salidroside and *p*-tyrosol from RCE.

Since adsorption chromatography exists, an intrinsic defect of separating compounds with close polarities, such as *p*-tyrosol, salidroside and the compound before salidroside (Figure 2G), was to be identified in Section 2.2.1; however, these three compounds cannot be separated by adsorption chromatography. This was validated by two other methods that were modified on the basis of the above method (data not shown). Therefore, alternative strategies based on different mechanisms should be utilized to separate those three compounds, thus preparing salidroside and *p*-tyrosol with high purity.

For adsorption chromatography, the material, polarity, pore diameter as well as the surface area of the resins were directly related to the capacities of adsorption and desorption for phenolics [29,30,31]. It was demonstrated that resins made from polystyrene as material showed better abilities of adsorption and desorption than those made from acrylate [29], and this finding made us choose resin made by polystyrene in the present work. In common polystyrene-made resins, non-polar resins are exhibited to be superior to weak-polar resins in respect to the adsorption and desorption of phenolics [29,30]. The resins with modest pore diameters showed optimal capacities of adsorption and desorption. This is due to the fact that large pore diameters can weaken the adsorption of molecules, while too small pore diameters can easily block the diffusion of adsorbent molecules [32]. Additionally, the surface area is another important factor that influences the abilities of the macroporous resins for the adsorption and desorption of phenolics [33]. A report has been made that resins with large surface areas exhibit much higher adsorption and desorption ratios [29]. Therefore, through a comprehensive comparison of the above aspects of common macroporous resins, DIAION HP-20 macroporous resins were finally chosen in this work, and it turned out that its effect is ideal in the enrichment of salidroside and *p*-tyrosol.

A report has been made that the targeted phenolics could be enriched in different elutions due to the different polarity of the solvent as eluent [30]. This is consistent with our results of the screen of eluent(s) for enrichment of the salidroside and *p*-tyrosol from RCE by DIAION HP-20 adsorption chromatography. Our results showed that ethanol concentrations ranged from 5–20% could effectively enrich salidroside and *p*-tyrosol, while ethanol concentrations greater than 25% and water could not. And the ratio of salidroside/*p*-tyrosol in the eluents notably changed with the increase of ethanol concentrations from 10% to 20%. Additionally, because the polarity of resin shifted with the change of the eluent, the phenolics’ concentration was distinctly different for each different elution [30,34], thus particular ethanol concentrations favor enriching certain phenol compounds when differed from polarity. Therefore, we finely investigated the effect of 5–20% ethanol on the enrichment of salidroside and *p*-tyrosol via a gradient elution. Our results showed that 5–13.33% ethanol fraction, namely, FEST (fraction enriching salidroside and *p*-tyrosol), made the best effect on the enrichment of these two compound.

### 2.2. Separation of Salidroside and p-Tyrosol from FEST by Silica Gel Chromatography

#### 2.2.1. Identification of the Compounds Occurred before Salidroside

**Figure 4:**
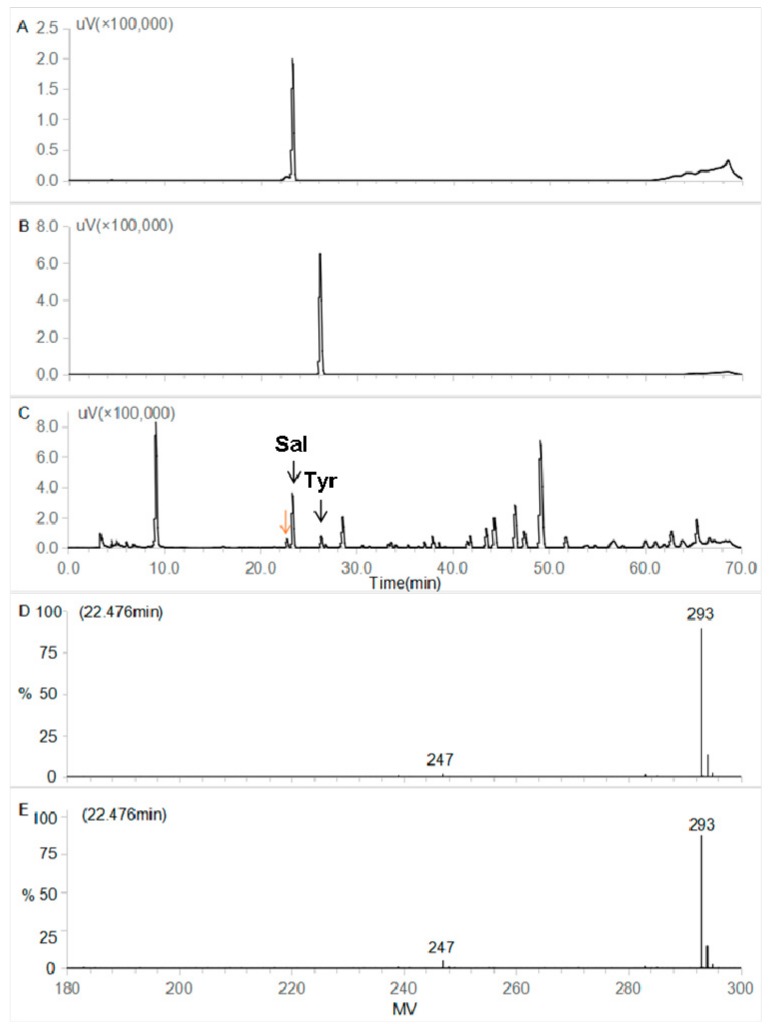
Liquid chromatographic profiles of salidroside, *p*-tyrosol and RCE, and ion fragment profiles of Q-TOF-MS presented at 22.476 min (yellow arrow). Shown in sequence are Liquid chromatographic profiles of salidroside (**A**), *p*-tyrosol (**B**) and *R. crenulata* crude extract (RCE) (**C**); ion fragment at 22.476 min by Q-TOF-MS-ES^−^ (D) and ion fragment at 22.476 min by Q-TOF-MS/MS-ES^-^ (**E**). Sal and Tyr refer to salidroside and *p*-tyrosol, respectively.

Before the screening of the optimal organic solvent(s) for the silica gel chromatography, the unknown compound accompanied with and occurred before the salidroside in Figure 2C,E,G was first identified by UPLC-Q-TOF-MS-ESI, after a desirable UPLC separation condition was developed (Figure 4C). As confirmed by their standards (Figure 4A,B), salidroside and *p*-tyrosol were observed at 23.242 and 26.119 min (Figure 4C), respectively. The unknown compound occurred before the salidroside was presented at 22.476 min. Through ion fragment analysis, a pseudo negative molecular ion [M + HCOOH − H]^−^ at *m*/*z* 293.1245 and a [M − H]^−^ at *m*/*z* 247.1199 was presented in both the Q-TOF-MS-ES^−^ and Q-TOF-MS/MS-ES^−^ ion fragment profiles (Figure 4D,E). This indicated that the molecular weight of the compound is 248, which was also in agreement with those values reported by Han et al. [35], thus it was identified as crenulatin (C_11_H_20_O_6_).

#### 2.2.2. Screen of Optimal Organic Solvent(s) for the Silica Gel Chromatography

In order to screen out optimal organic solvent(s) for the separation of crenulatin, salidroside, and *p*-tyrosol, several solvents including chloroform, ethyl acetate, saturated ethyl acetate, and saturated n-butanol were tested through a liquid-liquid extraction of aqueous FEST (see Figure 2G and Table 1). Saturated ethyl acetate and saturated n-butanol were commonly used in liquid-liquid extraction as separating solvents, and were different from those of their non-saturated counterparts in separation properties, thus being applied in this process.

As we saw in Table 2, extractions were divided into three classes, namely, four extractions with a single solvent, one successive extraction with ethyl acetate and chloroform, and two extractions with twice chloroform. Volume ratios of the above solvents to FEST were all set at 20:1 (*v*:*v*) to ensure full extraction, with an exception of one of the two extractions with twice chloroform being set at 40:1 (*v*:*v*).

The peak percentage was calculated as follows: (a certain peak area/total peak area in UPLC profile) × 100%. Comparison of peak percentages before (i.e., FEST) and after extraction explains changes of a certain peak by relevant extraction schemes. As shown in Table 2, peak percentages of salidroside extracted with each of individual organic solvents and the successive extraction at a ratio of 20:1 were all increased notably comparing to that in FEST, with the one extracted by single saturated n-butanol as an exception, whereas peak percentages of *p*-tyrosol all decreased, except crenulatin, which showed only slight elevations, suggesting that extractions with single chloroform, ethyl acetate, and saturated ethyl acetate, and the successive extraction possessed a certain capacity for separating salidroside from *p*-tyrosol and crenulatin. However, extraction with single saturated n-butanol was not suitable for this separation due to the simultaneous decrease in peak percentages of both the salidroside and *p*-tyrosol.

To confirm the results from the peak percentage, the distribution rate was further calculated to compare changes of a certain peak area before and after extraction according to the following formula: (a certain peak area from extraction schemes/the peak’s area of FEST (in water) × 100%. As we saw in Table 2, it is clear that the extraction with a single ethyl acetate and the successive extraction made the distribution rates of the salidroside increase by 14% and 18%, respectively. Extraction with a single chloroform made the distribution rate of the salidroside nearly constant (103%); but the extractions with a single saturated ethyl acetate and saturated n-butanol made their distribution rates decrease to 73% and 16%, respectively. Concerning the distribution rate of *p*-tyrosol, extractions reduced it to 53% with a single chloroform, 23% with a single ethyl acetate, 16% with a single saturated ethyl acetate, 12% with a single saturated n-butanol, and 13% with the successive ethyl acetate and chloroform. As to the distribution rate of the crenulatin, all extractions decreased it, with the extractions using chloroform of the single (103%) and the twice at a ratio to FEST of 40:1 (101%) were kept basically constant as two exceptions. Therefore, the above results demonstrate that the extractions with a single ethyl acetate and chloroform and the successive extraction can separate the two compounds while the extraction with a saturated n-butanol could not. This is consistent with the findings from the peak percentage. The difference existed in the extraction with a single saturated ethyl acetate. The result showed that the distribution rates of crenulatin, salidroside, and *p*-tyrosol all declined by extraction with a single saturated ethyl acetate, which is different from that of the peak percentage. This difference may be explained by the deduction that the increase in distribution rate of the salidroside by extraction with single ethyl acetate may be attributed to the transfer of water into the ethyl acetate during the liquid-liquid extraction rather than the capacity of the ethyl acetate itself.

**Figure 5:**
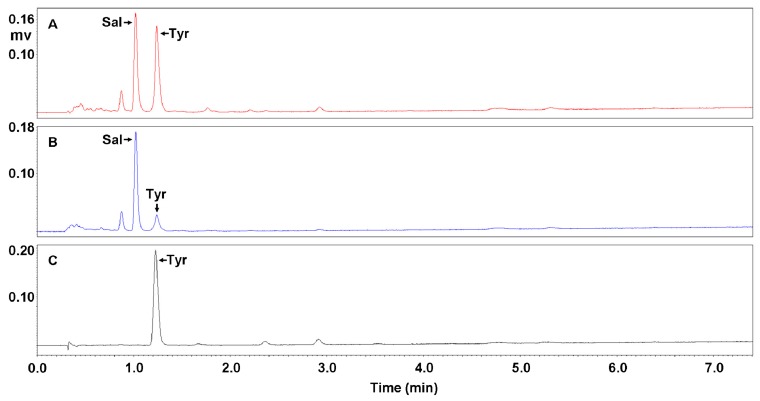
UPLC profiles of extraction with a single chloroform. Shown in sequence are profiles of fractions enriching salidroside and *p*-tyrosol (i.e., FEST in water) (**A**), aqueous FEST after extraction (**B**), and chloroform after extraction (**C**). Ordinates were proportionally adjusted for comparison. Sal and Tyr refer to salidroside and *p*-tyrosol, respectively.

Therefore, to clarify the above probability, the recovery rate was subsequently compared with the distribution rate to further evaluate the effects of the extraction schemes. It was calculated as followed: [(a certain peak area from extraction scheme × remaining aqueous FEST volume after extraction)/(the peak area of FEST (in water) × volume of FEST (in water)] × 100%. The results of the recovery rate and the distribution rate being the same suggest that there was no inter-solubility that existed between the organic solvent and the water, and vice versa. From Table 2, it can be observed that the extraction with a single ethyl acetate and the successive extraction made the recovery rates of both the salidroside and *p*-tyrosol decrease, while extraction with a single chloroform made no change when compared to the distribution rates. This implied that it was the ethyl acetate that made changes to the recovery rates of both the salidroside and *p*-tyrosol. Furthermore, because extraction with a single saturated ethyl acetate made the recovery rate not change when compared with the distribution rate, it is denoted that the inter-solubility did exist between the ethyl acetate and water. Therefore, the above deduction was confirmed, and a conclusion can be made that ethyl acetate is not capable of separating salidroside and *p*-tyrosol. The recovery rate also showed that the chloroform made no change in the recovery rates compared with the distribution rates, which was further confirmed by the extraction with a single chloroform two times, both at ratios of 20:1 and 40:1 (Table 2). This finding indicated that there is no water transfer in the extraction with a single chloroform during the liquid-liquid extraction, and a separation of the salidroside and *p*-tyrosol by extraction with a single chloroform (Figure 5) is due to the capacity of the chloroform itself.

Taken together, a conclusion can be made that the extraction with a single chloroform is an ideal method for the separation of salidroside and *p*-tyrosol (Figure 5B,C), thus chloroform was chosen as a solvent in the following silica gel chromatography to ensure a full separation of the salidroside and *p*-tyrosol by continuous elution.

#### 2.2.3. Simultaneous Separation of Salidroside and *p*-Tyrosol by the Silica Gel Chromatography

**Figure 6:**
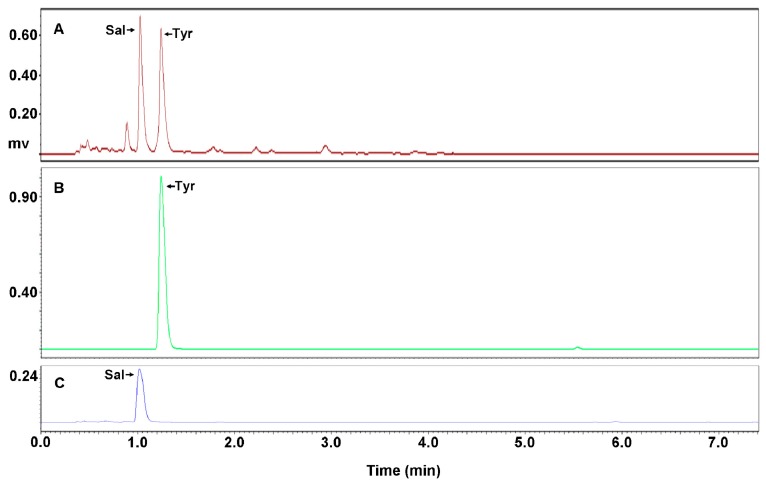
UPLC profile for isolation by silica column chromatography. Shown in sequence are profiles of fraction enriching salidroside and *p*-tyrosol (i.e., FEST) (**A**), *p*-tyrosol (**B**), and salidroside (**C**). Ordinates were proportionally adjusted for comparison. Sal and Tyr refer to salidroside and *p*-tyrosol, respectively.

For fully separating crenulatin, salidroside, and *p*-tyrosol from each other and obtaining salidroside and *p*-tyrosol with high purity from FEST (Figure 6A), silica gel chromatography was then adopted based on the above findings from the liquid-liquid extraction. As Figure 6B shows, *p*-tyrosol was fully isolated from salidroside by continuous elution for one bed volume of chloroform. For further separation of the salidroside from the crenulatin, a mixed solvent by chloroform with methanol at a volume ratio of 4:1 was found, and the optimum was reached when four bed volumes were used (Figure 6C). A successive elution procedure was thus established by elution with chloroform for one bed volume followed with a mixture of chloroform and methanol at a volume ratio of 4:1 for four bed volumes. And it was also exhibited by Table 3 that salidroside and *p*-tyrosol reached concentrations of 94.17% and 97.29%, from those of 31.44% and 36.80% in FEST, with an impressive rate increase of 62.73% and 60.48%, respectively, indicating that high purities of salidroside and *p*-tyrosol were successfully prepared out from FEST by silica gel chromatography. A statistical difference between concentrations of salidroside and *p*-tyrosol before and after silica gel chromatography indicated that the above procedure by silica gel chromatography was notably effective for the separation of salidroside and *p*-tyrosol, as well as crenulatin (Table 3).

The solubility of the phenolics is governed by their chemical nature, which varies from their different structures, such as extent of polymerization, number, as well as position of hydroxyl groups or sugar moieties. The solubility of the phenolics is also influenced by the polarity of the solvent [36,37]. Methanol, ethanol, acetone, water, ethyl acetate, and their combinations are frequently used for the extraction of phenolics, and their effects on the constituents and amount of phenolic compounds being extracted are varied [36]. Ren et al. [38] compared the phenolic constituents being extracted and the extraction efficiency by four organic solvents (ethyl acetate, chloroform, petroleum ether and hexane). It was demonstrated that ethyl acetate extracted approximately all phenolics and its extraction efficiency was close to 100%. It was slightly higher than chloroform, followed by petroleum ether, and hexane, which exhibited a low effect as well as efficiency. Due to a minor difference in polarity between crenulatin, salidroside, and *p*-tyrosol demonstrated by three very close peaks in the UPLC profile, it is difficult to separate these three compounds. Therefore, in order to overcome this difficulty, we investigated the effects of various organic solvents on the separation of these three compounds, concerned through a liquid-liquid extraction of aqueous FEST. Chloroform, ethyl acetate, saturated ethyl acetate, and saturated n-butanol were examined. After a comparison of the peak percentage, distribution rate, and recovery rate of the above examined organic solvents, chloroform was demonstrated to be capable of separating salidroside and *p*-tyrosol. Furthermore, separation completeness by chloroform was proved to be enhanced with the increase in the extraction times. Therefore, in order to realize the full separation of salidroside and *p*-tyrosol, a continuous elution of chloroform was then adopted via silica gel chromatography. For the separation of salidroside from crenulatin, we carefully compared different combinations of chloroform and methanol, and a solvent prepared by chloroform mixed with methanol at a volume ratio of 4:1, which proved to be optimal in the silica gel chromatography. Therefore, a method by silica gel chromatography was finally established for the simultaneous separation of crenulatin, salidroside, and *p*-tyrosol.

### 2.3. Recovery and Yields

The results of the recovery rate are summarized in Table 4. As shown in Table 4, in the first process, namely the HP-20 adsorbent resin, FEST simultaneously concentrated salidroside and *p*-tyrosol from RCE with the concentrations reaching 31.44% and 36.80%, respectively, and their recovery rate was achieved at 64.97% and 50.51%, correspondingly. In the following process of silica column chromatography, 94.17% purity of salidroside and 97.29% of *p*-tyrosol were obtained, and their recoveries were 62.60% and 89.58%, respectively. Furthermore, an overall yield of salidroside was 39.09%, and that of *p*-tyrosol reached 43.73%.

Li and Chen [39] reported that salidroside alone was successfully prepared using a high-speed counter-current chromatography with a purity of 98%; however, their yield was only 12.8%. Ma et al. [27] developed a two-step preparation process simply for salidroside as well by using adsorption chromatography. The yield reached to 48.82%, although the purity was 92.21%. The present study developed the two-step method that can simultaneously prepare salidroside and *p*-tyrosol both with higher purities as well as higher overall yields.

## 3. Materials and Methods

### 3.1. Preparation of Rhodiola Crude Extracts (RCE)

*Rhodiola crenulata* (Hook. f. et Thoms.) H. Ohba was purchased from Beijing Tongrentang Pharmacy (Beijing, China) and authenticated as rhizome of *R. crenulata* by associate professor Dr. Zhonghua Liu at the Beijing Forestry University. For the standards, salidroside was purchased from the National Institute for the Control of Pharmaceutical and Biological Products, Beijing, and *p*-tyrosol was bought from Sigma-Aldrich Chemical Co., Beijing, China. Prior to extraction, rhizome of *R. crenulata* was first ground into a fine powder using a mortar and pestle. For every 5 g of fine powder, 100 mL of petroleum ether was added and the mixture sonicated in a water bath for 30 min at room temperature, in order to remove lipid-soluble impurities. The sonicated mixture was then centrifuged at 1500× *g* for 10 min and the supernatant removed. The remnant was air-dried and kept for continuing extraction. Briefly, 100 mL of 75% methanol was added to the remnant and the mixture was sonicated for 30 min with occasional stirring, followed by centrifugation at 1500× *g* for 10 min and supernatant collection. These steps were performed two more times, and the collected supernatants were pooled and transferred to a rotary evaporator (Laborota 4011, Heidolph, Germany) to remove any methanol. The remaining aqueous portion was stored at 4 °C overnight, and then centrifuged at 1500× *g* for 15 min to collect the supernatant. This supernatant water solution was named as *rhodiola* crude extract (RCE), which was then loaded onto the adsorption column for initiative purification.

### 3.2. Purification by Adsorption Chromatography to Obtain Fraction Enriching Salidroside and p-Tyrosol (FEST)

Dry DIAION HP-20 adsorbent resin (Mitsubishi, Tokyo, Japan) of 100 g was first pretreated by soaking in 50% ethanol at 4 °C overnight, and followed by washing with water in the Buchner funnel till no alcohol remained. After full adsorption to substances within the RCE, the HP-20 resin slurry was then loaded into a column (30 cm height and 1.5 cm radius) to about 2/3 height with a bed volume (BV) of about 150 mL. Subsequently, the column was eluted with 2 BVs of water, followed by 5%, 10%, 15%, 20%, 25%, and 50% aqueous ethanols in sequence (each for 1 BV). Then concentrations of salidroside and *p*-tyrosol in each eluent were examined by UPLC, and the optimal eluent(s) for enrichment of salidroside and *p*-tyrosol from RCE were screened.

Based on the finding by the screen of optimal eluent(s), a method for enrichment of salidroside and *p*-tyrosol from RCE by DIAION HP-20 adsorption chromatography was established. In brief, after washing with 5 BVs of water and 2 BVs of 5% ethanol in sequence, the column was subjected to 3 BVs of gradient elution from 5% to 20% ethanol. In the process of gradient elution, 180 elutions, each containing 2.5 mL of eluent, were collected using a Waters III fraction collector (Waters Corporation, USA) equipped with an Econo-Column pump (BIO-RAD, Japan) regulating a flow rate at 1.2 mL/min. Elutions No. 1–100 (i.e., fraction enriching salidroside and *p*-tyrosol (FEST)) were pooled, which corresponded to 5–13.33% ethanol. FEST was then stored in 4 °C for liquid-liquid extraction to screen of optimal organic solvent(s), as well as further separation of salidroside and *p*-tyrosol by silica gel chromatography.

It is noted that at the end of each of the above purification methods, the column was regenerated by washing with 50% ethanol (1 BV), followed by 100% for 3 BVs.

### 3.3. Liquid-Liquid Extraction to Screen Optimal Organic Solvent(s) for Silica Gel Chromatography

The ethanol in FEST from DIAION HP-20 adsorption column was firstly removed by evaporation with reduced pressure. The aqueous FEST was then divided into two portions, among which a portion of the aqueous FEST was then placed in a separating funnel to extract by different solvents at a ratio of 20:1 (solvents:aqueous FEST, *v*:*v*), including chloroform, ethyl acetate, saturated ethyl acetate and saturated n-butanol. In addition, a successive extraction with ethyl acetate and chloroform was also performed. For the extraction with chloroform, twice extractions were performed at volume ratios of 20:1 and 40:1. Subsequently, the aqueous FEST after extraction was subjected to UPLC for analyzing salidroside and *p*-tyrosol.

### 3.4. Isolation of p-Tyrosol and Salidroside by Silica Gel Chromatography

Another portion of the above aqueous FEST was stored in −80 °C for 3 h, and then freeze-dried into powder using a vacuum freeze dryer. Subsequently, it was dissolved in 2 mL of 75% methanol, and subjected to chromatography (40 cm × 1 cm, loading BV = 100 mL) on a column of silica gel (20 g, 200–300 mesh) and successively eluted with chloroform (1 BV) and mixture of chloroform and methanol (*v*:*v* = 4:1, 4 BVs) to afford eluates. Fifty 5-mL elutions were collected by the fraction collector by setting a flow rate at 2 mL/min. Elutions were then evaporated with reduced pressure to dryness, dissolved in 75% methanol, and injected into UPLC for analysis. Elutions No. 7–8 and No. 24–31 were combined, in which *p*-tyrosol and salidroside were included, respectively.

### 3.5. UPLC and UPLC-Q-TOF-MS Analyses

UPLC analysis were performed using a Waters UPLC system equipped with a PDA eλ detector, a binary solvent manager, and a sample organizer controlled by analytical software (Empower 2.0, ACQUITY^TM^ Ultra Performance LC, Waters, Milford, MA, USA). The chromatographic separation was performed on a reversed phase column (ACQUITY UPLC BEH C_18_ 50 mm × 2.1 mm i.d., 1.7 μm, Waters, Milford, MA, USA) with column temperature set at 28 °C. Two solvents were applied for elution: water containing 0.1% (*v*:*v*) acetic acid (A) and methanol (B). A linear gradient program was adopted for UPLC separation: 1–7 min, 15–40% B; 7–8 min, 40–100% B; 8–9.1 min, 100–15% B; 9.1–12 min, 15% B. The flow rate was set at 0.4 mL/min, and the injection volume was 2 μL. The detection wavelength was set at 280 nm to monitor salidroside and *p*-tyrosol simultaneously. Each extract was filtered through a 0.22-μm nylon filter and analyzed under the above UPLC separation. Peak identifications were conducted by matching the retention times with those of standards.

Identification of salidroside, *p*-tyrosol and the unknown compound presented before salidroside was conducted using the Acquity UPLC system coupled to a Xevo-G2QTOF mass spectrometer (Waters, Milford, MA, USA) with an ESI interface operating in negative ion resolution mode under a capillary voltage of 2.5 kv. The desolvation gas flow rate was set at 11.6 L/min, and the temperature for desolvation was 280 °C. A mass range of 50–1200 *m*/*z* was selected. UPLC-MS/MS data, including retention time, experimental and calculated *m*/*z*, molecular formula, error of the experimental *m*/*z*, DBE, and MS/MS fragments, were collected. The chromatographic separation was performed on an Acquity Ultraperformance Liquid Chromatography (UPLC) system (Waters, USA) equipped with a Dimonsil C_18_ column (5 μm, 4.6 mm × 250 mm, Dikma Technologies, Lake Forest, CA, USA). Extract and standards were filtered through the 0.22-μm filter before injection. Column temperature was maintained at 28 °C and injection volume was set at 10 μL. Mobile phase consisted of water-0.5% formic acid (A) and acetonitrile-0.5% formic acid (B). The gradient program was as followed: 0–10 min, 5%B; 10–25 min, 5–12%B; 25–35 min, 12–18%B; 35–55 min, 18–22%B; 55–65 min, 22–28%B; 65–75 min, 28–5%B; 75–85 min, 5%B. Elution was set at a flow rate of 0.8 mL/min, wavelength was 280 nm. Identification of salidroside, *p*-tyrosol and the unknown compound presented before salidroside from the extract was based on the data in comparison with those of the standards and literatures.

### 3.6. Statistical Analysis

Data were presented as mean ± SD of three replicates. The statistical significance was determined by using Microsoft Excel statistical software (Microsoft Office Excel 2016, Microsoft Corp., Redmond, WA, USA). A *t*-test (two-sample equal variance and two-tailed distribution) was applied, and *p* < 0.05 was set to be significant.

## 4. Conclusions

The *Rhodiola* species is a traditional medicine in Asian and eastern European countries with various pharmacological properties. Salidroside and *p*-tyrosol are well accepted as their main responsible compounds, and commonly used as quality control markers. In the present work, we have developed an UPLC-guided two-step method for simultaneous preparation of salidroside and *p*-tyrosol from rhizome of *R. crenulata*.

The main findings of this method are summarized as follows: (1) The two-step method can be used for the simultaneous preparation of high purities of salidroside (94.17%) and *p*-tyrosol (97.29%) from *R. crenulata* with an overall yield of 39.09% for salidroside and 43.73% for *p*-tyrosol. This offers valuable information regarding the preparation of these two compounds from other *Rhodiola* and even other related plant species; (2) DIAION HP-20 is an ideal macroporous resin for enriching salidroside and *p*-tyrosol under the gradient elution from 5% to 13.33% ethanol; (3) Chloroform is ideal for further separation of salidroside and *p*-tyrosol, and mixed solvent by chloroform and methanol at a volume ratio of 4:1 can separate salidroside with crenulatin via silica gel chromatography. Therefore, this method offers a novel way for simultaneous preparation of salidroside and *p*-tyrosol with high purities as well as noticeable overall yields from rhizome of *R. crenulata*. The present method might contribute to the future development of *R. crenulata* and other *Rhodiola* species, as well as its utilization in the pharmaceutical industry.

## Figures and Tables

**Table 1:** Effects of DIAION HP-20 adsorbent resin on purification of salidroside and *p*-tyrosol.

	Concentration (%)		Increasing Rate (%)	
	Salidroside	*p*-Tyrosol	Salidroside	*p*-Tyrosol
RCE *	2.25 ± 0.13	3.36 ± 0.13		
15% Ethanol eluent	21.51	20.28	19.26	16.92
FEST	31.44 ± 2.68 ^a^	36.80 ± 0.51 ^a^	29.19 ± 2.72	33.44 ± 0.63

In each row ^a^ letter means significant differences between two groups (*p* < 0.05). * RCE, R. crenulata crude extract; FEST, fraction enriching salidroside, and *p*-tyrosol.

**Table 2:** Influence of various combinations of solvents on separation of crenulatin, salidroside, and *p*-tyrosol by liquid-liquid extraction (% in water).

	Peak Percentage				Distribution Rate			Recovery Rate	
	Cre	Sal	Tyr	Others	Cre	Sal	Tyr	Sal	Tyr
FEST (in water)	8.38	33.76	36.27	21.59	100	100	100		
Extraction schemes									
C(20:1)1X	10.41	41.72	23.14	24.73	102	103	53	103	53
EA(20:1)1X	10.82	56.42	11.77	21.00	93	114	23	55	11
SEA(20:1)1X	10.94	54.57	12.93	21.56	59	73	16	73	16
Sn-B(20:1)1X	19.55	31.17	25.49	23.79	41	16	12	16	12
EA(20:1)1X/C(20:1)1X	9.92	59.66	6.97	23.45	79	118	13	47	5.2
C(20:1)2X	11.28	60.75	15.21	12.76	72	96	22	96	22
C(40:1)2X	11.26	49.70	10.14	28.90	101	110	21	110	21

Note: Sal, salidroside; Tyr, *p*-tyrosol; CRE: crenulatin; X, times; C, chloroform; EA, ethyl acetate; SEA, saturated ethyl acetate; Sn-B, saturated n-butanol; FEST, fraction enriching salidroside, and *p*-tyrosol; Data from FEST (in water) means data collected from aqueous FEST before extraction, while data from the extraction schemes is referred to as data collected from aqueous FEST after extraction.

**Table 3:** The effect of silica column chromatography on isolation of salidroside and *p*-tyrosol.

	Concentration (%)		Increasing Rate (%)	
	Salidroside	*p*-Tyrosol	Salidroside	*p*-Tyrosol
FEST *	31.44 ± 2.68	36.80 ± 0.51		
Salidroside	94.17 ± 3.16 ^a^	—	62.73 ± 5.20	—
*p*-Tyrosol	—	97.29 ± 2.37 ^a^	—	60.48 ± 2.55

In each row ^a^ letters means significant differences between two groups (*p* < 0.05). * FEST, fraction enriching salidroside and *p*-tyrosol.

**Table 4:** The recovery rate of salidroside and *p*-tyrosol through two step separation.

		**Ts**	**Ws**	**Wt**	**Ps**	**Pt**	**Rs**	**Rt**
		**(mg)**			**(%)**			
HP-20 adsorbent resin	Load	876.7	19.7	29.5	2.25	3.36	100	100
	FEST	40.6	12.8	14.9	31.44	36.80	64.97	50.51
Silica column chromatography	Load	39.1	12.3	14.4	31.44	36.80	100	100
	Salidroside	8.2	7.7	—	94.17	—	62.60	
	*p*-Tyrosol	13.3	—	12.9	—	97.29		89.58

* FEST, fraction enriching salidroside and *p*-tyrosol; Ts = total solids; Ws = weight of salidroside; Wt = weight of *p*-tyrosol; Ps = purity of salisroside; Pt = purity of *p*-tyrosol; Rs = recovery of salidroside; Rt = recovery of *p*-tyrosol.
